# PD37 Development Of A Tool For Quality Assessment Of Health Economic Evaluations

**DOI:** 10.1017/S0266462324003003

**Published:** 2025-01-07

**Authors:** Nayê Balzan Schneider, Celina Borges Migliavaca, Cinara Stein, Débora Dalmas Gräf, Gabrielle Nunes Escher, Sérgio Decker, Maicon Falavigna, Carisi Polanczyk

## Abstract

**Introduction:**

Health economic analyses compare the necessary investments and health outcomes for two or more technologies, assisting in resource allocation. How these analyses are conducted directly affects the results obtained. Therefore, it is essential to consider their quality during decision-making. The aim of this study was to develop a domain-based tool for the critical assessment of cost-effectiveness and cost-utility studies.

**Methods:**

We conducted a scoping review to identify tools available for the critical assessment of health economic analyses and extracted their recommendations. Based on the tools’ items and the discussions of a working group, we identified domains related to the methodological quality of health economic analyses for inclusion in the new tool. The items extracted during the scoping review were classified according to the previously defined domains and were used to identify complementary aspects that should be included in the new tool.

**Results:**

We identified 21 tools, all of which were checklists containing seven to 80 items. The following four quality domains were established for the new tool: (i) applicability of the research question; (ii) model structure; (iii) model parameters; and (iv) precision of the results. Assessment of each domain was guided by signaling questions. The first domain assessed the applicability of the research question to the desired setting; the second evaluated whether the model adequately represents the complexity of the clinical condition; the third assessed the quality (certainty) of the key parameters used in the model; and the fourth evaluated the certainty of the incremental cost-effectiveness or cost-utility ratio.

**Conclusions:**

The tool was developed to integrate critical aspects that affect the methodological quality of health economic analyses, which are often missing in other tools. The quality of reporting was not included as a domain because it is already covered by existing tools. A multidisciplinary panel with different key stakeholders is being organized to review and refine the first version of the tool.

